# A Web-Based Social Network Tool (GENIE) for Supporting Self-management Among High Users of the Health Care System: Feasibility and Usability Study

**DOI:** 10.2196/25285

**Published:** 2021-07-12

**Authors:** Ruta Valaitis, Laura Cleghorn, Ivaylo Vassilev, Anne Rogers, Jenny Ploeg, Anita Kothari, Cathy Risdon, James Gillett, Dale Guenter, Lisa Dolovich

**Affiliations:** 1 School of Nursing McMaster University Hamilton, ON Canada; 2 Department of Family Medicine McMaster University Hamilton, ON Canada; 3 School of Health Sciences University of Southampton Southampton United Kingdom; 4 School of Health Studies Western University London, ON Canada; 5 Health Aging and Society McMaster University Hamilton, ON Canada

**Keywords:** web-based tool, usability, feasibility, self-management, social network, primary care, health and social services, linkages, high systems users, volunteers

## Abstract

**Background:**

Primary care providers are well positioned to foster self-management through linking patients to community-based health and social services (HSSs). This study evaluated a web-based tool—GENIE (Generating Engagement in Network Involvement)—to support the self-management of adults. GENIE empowers patients to leverage their personal social networks and increase their access to HSSs. GENIE maps patients’ personal social networks, elicits preferences, and filters local HSSs from a community service directory based on patient’s interests. Trained volunteers (an extension of the primary care team) conducted home visits and conducted surveys related to life and health goals in the context of the Health TAPESTRY (Teams Advancing Patient Experience: Strengthening Quality) program, in which the GENIE tool was implemented. GENIE reports were uploaded to an electronic medical record for care planning by the team.

**Objective:**

This study aims to explore patients’, volunteers’, and clinicians’ perceptions of the feasibility, usability, and perceived outcomes of GENIE—a tool for community-dwelling adults who are high users of the health care system.

**Methods:**

This study involved 2 primary care clinician focus groups and 1 clinician interview (n=15), 1 volunteer focus group (n=3), patient telephone interviews (n=8), field observations that captured goal-action sequences to complete GENIE, and GENIE utilization statistics. The patients were enrolled in a primary care program—Health TAPESTRY—and Ontario’s Health Links Program, which coordinates care for the highest users of the health care system. NVivo 11 (QSR International) was used to support qualitative data analyses related to feasibility and perceived outcomes, and descriptive statistics were used for quantitative data.

**Results:**

Most participants reported positive overall perceptions of GENIE. However, feasibility testing showed that participants had a partial understanding of the tool; volunteer facilitation was critical to support the implementation of GENIE; clinicians perceived their navigation ability as superior to that of GENIE supported by volunteers; and tool completion took 39 minutes, which made the home visit too long for some. Usability challenges included difficulties completing some sections of the tool related to medical terminology and unclear instructions, limitations in the quality and quantity of HSSs results, and minor technological challenges. Almost all patients identified a community program or activity of interest. Half of the patients (4/8, 50%) followed up on HSSs and added new members to their network, whereas 1 participant lost a member. Clinicians’ strengthened their understanding of patients’ personal social networks and needs, and patients felt less social isolation.

**Conclusions:**

This study demonstrated the potential of GENIE, when supported by volunteers, to expand patients’ social networks and link them to relevant HSSs. Volunteers require training to implement GENIE for self-management support, which may help overcome the time limitations faced by primary care clinicians. Refining the filtering capability of GENIE to address adults’ needs may improve primary care providers’ confidence in using such tools.

## Introduction

### Background

It has been reported that globally, 1 in 3 adults have multiple chronic conditions (MCCs) [1]. There was an increase from 45.7% in 1988 to 59.6% in 2018 with regard to adults in the United States with 2 or more MCCs, and the weighted prevalence of 2 or more MCCs was higher in those aged ≥65 years [2]. Fostering self-management support for health conditions is particularly important, given the rising numbers and projected rise in complex multimorbidity. Self-management support builds problem-solving skills to enhance self-efficacy to carry out behaviors toward a desired goal and can support positive health outcomes, reduce the burden of long-term conditions for the patient and the health system, and decrease health system costs [3,4]. A qualitative systematic review identified challenges that patients experienced with self-management, including dealing with physical and emotional symptoms; living with pain, depression, and fatigue; and having a lack of understanding of self-management strategies related to conflicting information from providers [5]. Kang et al [6] found that quality of life scores were higher among patients with good versus low self-management strategy scores regardless of the number of comorbidities.

Improving access to health and social services (HSSs) to address self-management can be supported through information, referrals, facilitation, and system navigation by primary care providers [7-9]. Results from a longitudinal study of 300 randomly selected patients with diabetes or chronic heart disease found that connecting people to social support resources, including a variety of people and groups, supported self-management and physical and mental health [10]. Patients with multiple and complex health and social conditions are likely to derive the maximum benefit from linkages to HSSs [11].

In recent years, researchers have established that social networks can influence positive health behaviors and practices, and this is also true in populations that are managing long-term conditions [12-15]. Social connectedness has been shown to be particularly beneficial for vulnerable groups, such as those living in poverty and with chronic illnesses [16]. Reeves et al [10] established associations between connections with and the use of local networks, resulting in improved physical and mental well-being and better coping with their conditions. Personal and social networks and relationships in community settings can act as a conduit for accessing resources and provide support for managing long-term conditions, which can complement what is provided by formal service provision.

The implementation of a self-management support intervention in 31 primary care settings in England had poor uptake because of a perceived lack of relevance and fit to accessible sources of support and because primary care health care professionals did not prioritize self-management support [17]. Primary care providers in Canada have been tasked with fostering self-management through support and coaching, referral management, and linking to relevant community-based resources and services [4,18,19]. However, like their UK peers, they have struggled with limited time for coaching; a lack of knowledge of what community-based HSSs are available and how they can address health and social needs; and a lack of time to keep up with changing community services, including concerns about their quality [20,21]. Despite these challenges, it is argued that there is a need for primary care providers to implement effective self-management support interventions that incorporate connections to community resources for those living with long-term conditions [22] and, particularly, for those who are known to be isolated, requiring more encouragement to make connections [23-25].

The aim of the GENIE (Generating Engagement in Network Involvement) tool was to encourage the expansion of a patient’s social networks to reduce the negative health impacts of long-term conditions and to reduce the concomitant social effects, such as social isolation and loneliness [23]. GENIE is a web-based tool that aims to support self-management by leveraging adults’ engagement with their personal social network to facilitate the uptake of relevant community-based activities and HSSs. Studies have shown that when GENIE was delivered by trained facilitators to adults with chronic health and social conditions in the community settings, there was an increase in the diversity of participants’ networks and greater engagement with community activities [26,27]. Given these positive results and the challenges faced by primary care providers in implementing self-management strategies [17], research is needed to understand the feasibility, usability, and perceived impacts of implementing GENIE within the primary context, with the use of trained facilitators as an extension of the primary care team. This knowledge will be useful to inform future implementation of the GENIE tool in primary care and can serve as a basis for the development of outcome measures to be used in future controlled studies.

### Research Questions

This study examined the feasibility, usability, and perceived patient outcomes of the implementation of GENIE with adults enrolled in Ontario’s Health Links Program. This program alerts health providers to individuals with high rates of health service utilization to target care and thereby reduce health care costs [28]. The research questions were as follows:

1. What is the usability and feasibility of implementing GENIE, facilitated by lay volunteers and primary care providers, with 55- to 69-year-old adults enrolled in the Health Links Program?
2. What are patients’, providers’, and volunteers’ perceptions of the impact of the use of GENIE?

## Methods

### Social Network Tool (GENIE)

#### Overview

**Figure 1:**
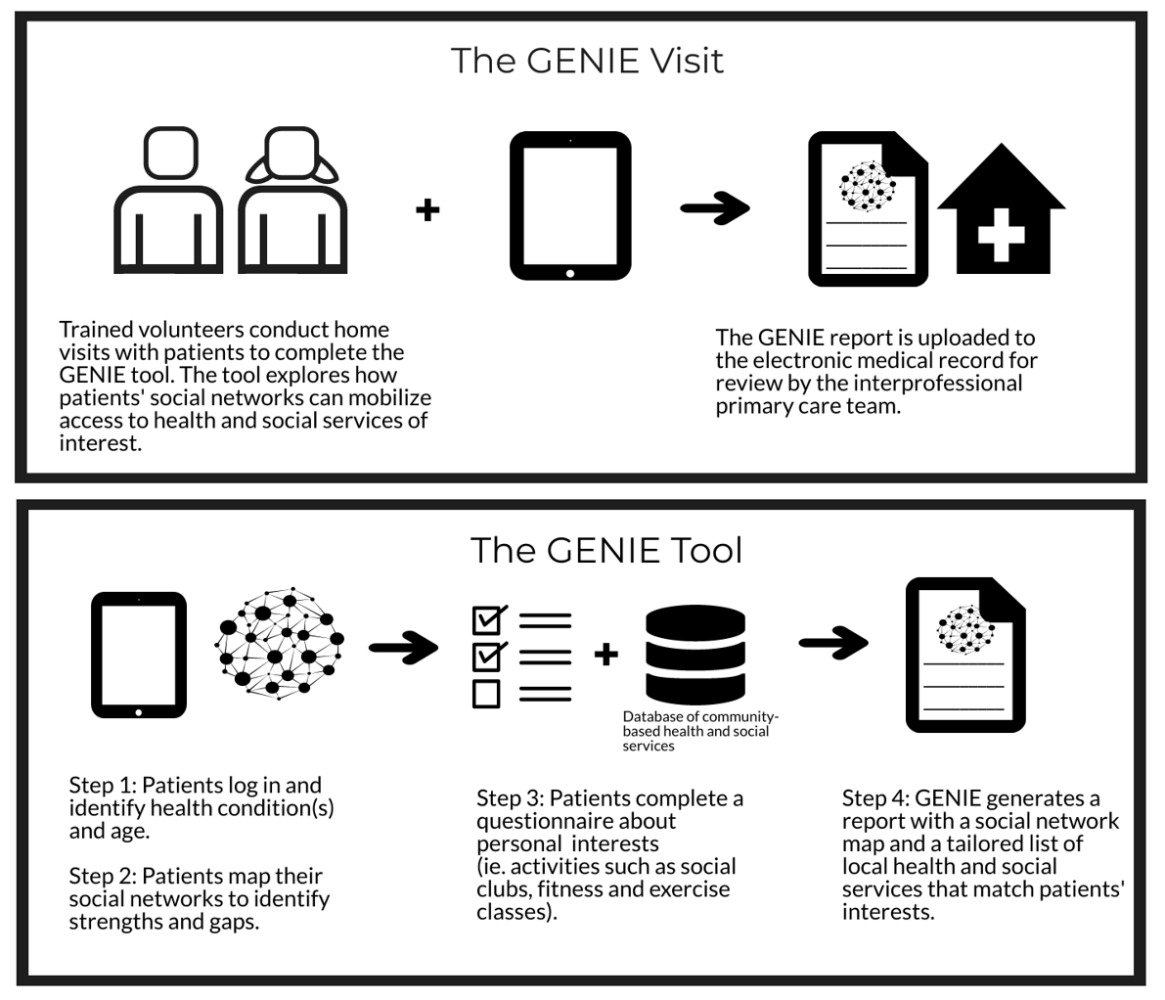
Depiction of the GENIE visit and tool. GENIE: Generating Engagement in Network Involvement.

GENIE is a web-based tool designed by a team of researchers from the United Kingdom [29]. GENIE has been previously implemented by trained lay or health care workers in various contexts in the United Kingdom and Europe to link patients to community-based HSSs to support them in reaching their life and health goals [22,24,26]. The GENIE tool has 3 core functions: (1) mapping a patient’s personal social network to better understand a patient’s support network and identify possible network members who can assist them; (2) selecting topics of interest that relate to patients’ interests under the categories of activities, health, learning, support, independent living, volunteering, and pets; and (3) geolocating local community programs, services, and resources related to the selected categories (Figure 1).

The GENIE tool consists of 4 steps. First, patients enter an email address (or get help to obtain an email address) to log in and enable them to save their results, select from a list of common health conditions (eg, heart problems, stroke, diabetes, arthritis, and cancer), and enter their age and postal code.

**Figure 2:**
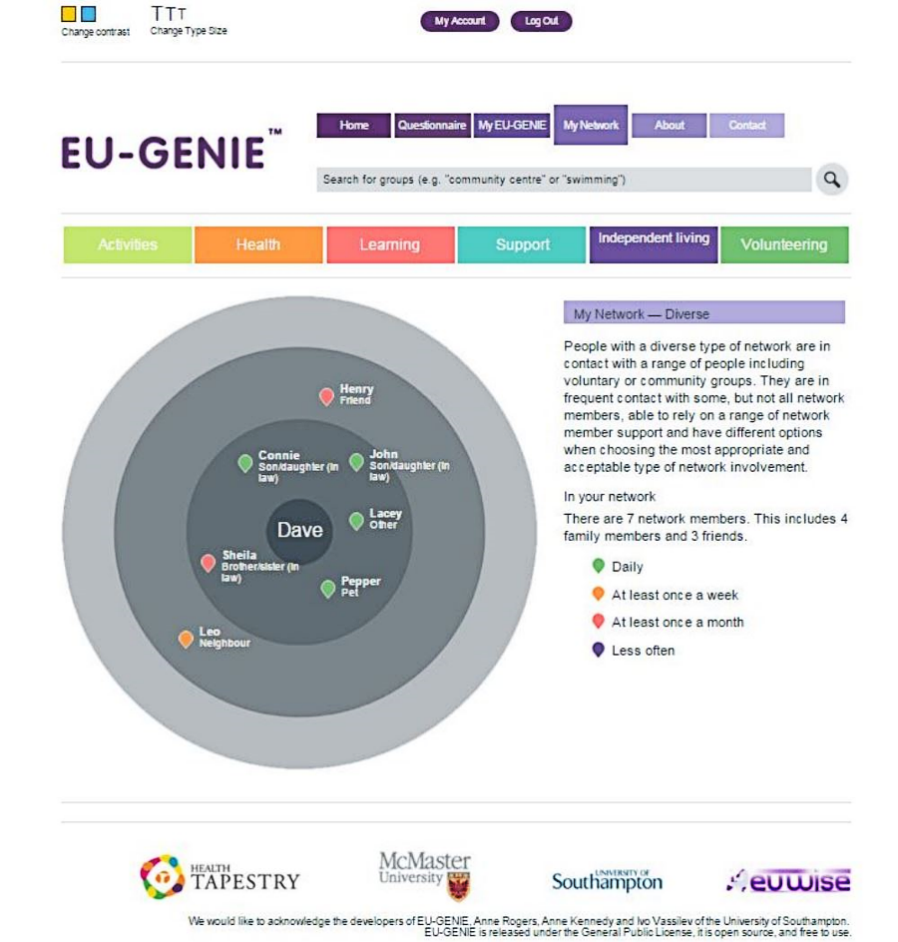
Example of network mapping and categories of interest in GENIE (Generating Engagement in Network Involvement).

Second, the patients generate a personal network map that lists individuals, groups, or organizations (eg, son or daughter-in-law, friend, and social club) that the patients consider important to them in relation to being healthy and living at home. Network members who are deemed to be most important to them are placed closest to the center of the circle, where the patients are placed, with others moving out into the outer circles. Each member is categorized by type (eg, family, friend, neighbor, group, or organization), which determines the typology of that patient’s network. Network typologies can consist of mostly friends and family members; mostly professionals; or a diverse mix of professionals, organizations, friends, and family (eg, *My Network–Diverse*; Figure 2). Diverse networks are the most robust social networks in the GENIE typology containing family, friends, and *weak tie* relationships, whereas *very isolated* and *friend and family supported* networks have fewer members and less diversity of relationships. Research has shown that people with long-term health conditions and diverse networks are associated with enhanced self-management skills [30]. Patients also indicate the frequency at which they meet with each network member. This information can help identify network members who may be more available to support a patient’s HSS use.

Third, patients answer 12 questions about their interests organized under the following categories: (1) activities, (2) health, (3) learning, (4) support, (5) independent living, (6) volunteering, and (7) pets. Some questions have subquestions, for example, if a patient is interested in *activities*, they are prompted with subquestions to refine the topic (ie, reading and writing, drama and music, arts and crafts, or social clubs).

**Figure 3:**
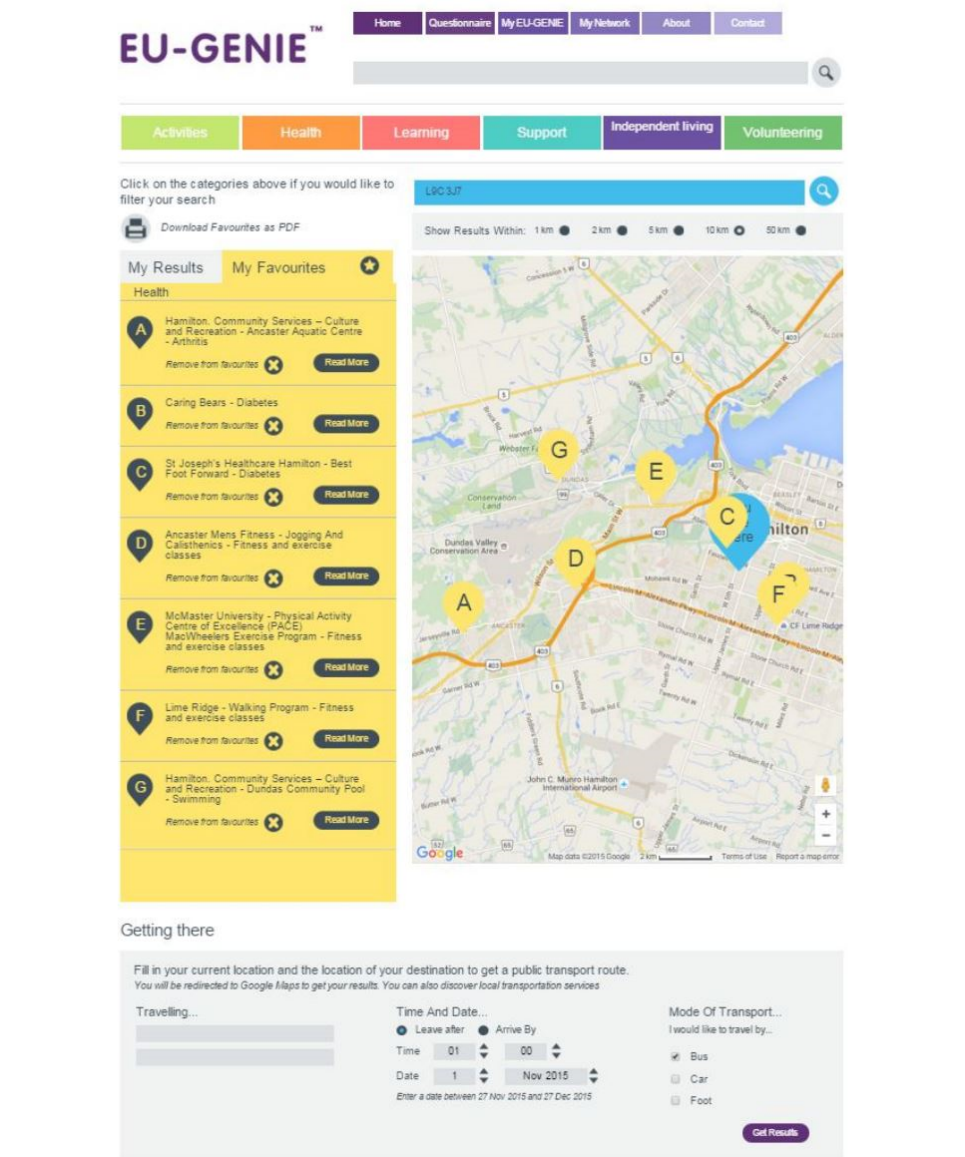
Example of a user’s favorited list of links geomapped for the health category. GENIE: Generating Engagement in Network Involvement.

Fourth, once the patients complete the questionnaire, they move to a web page listing links to relevant community-based HSSs organized under the relevant categories. For example, if a patient indicates that they are interested in physical activity, they can find HSSs listed under the *health* tab related to this subactivity (Figure 3). HSSs are geomapped for selection based on the patient’s preferred distance from their postal code (1 km, 2 km, 5 km, 10 km, or 50 km). Relevant HSSs, including a brief description of their programs or services, are populated from the region’s community information database.

**Figure 4:**
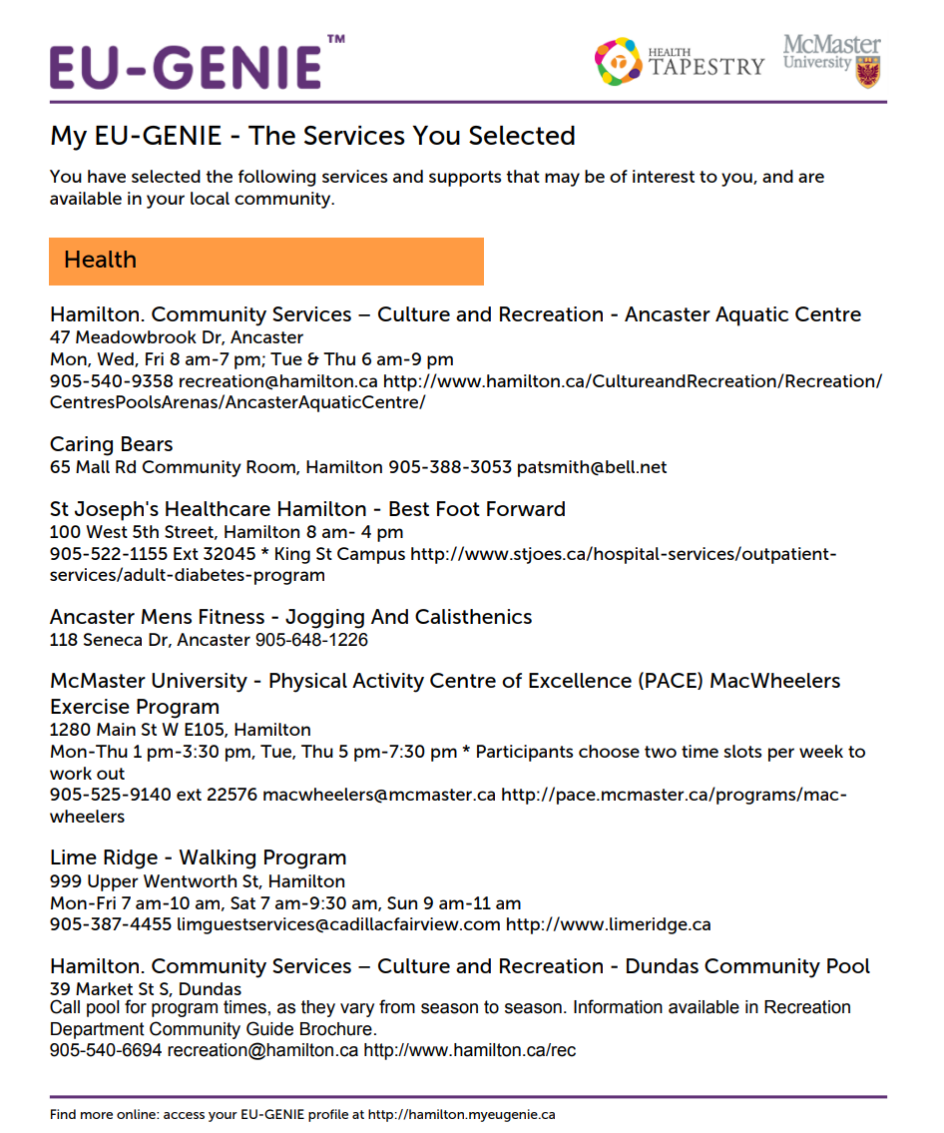
An example of a printed report of favorites for users to follow up. GENIE: Generating Engagement in Network Involvement.

Patients review their results and mark their *favorites* (Figure 3), which can be saved, downloaded, and printed in a short report for easy access (Figure 4) [13]. Facilitators were to encourage patients to consider their social networks to help in overcoming barriers to access the desired HSSs.

#### Adaptation of GENIE for Use in Canada

GENIE was adapted for use in Hamilton, Canada. The UK open-source tool required minor word modifications in the questionnaire to address the linguistic differences between Canadian and British audiences (eg, changing the word *befriending* to *friendly visiting*). A second significant adaptation was linking GENIE to a back-end database of community-based HSSs. In the United Kingdom, community information services with databases, such as those in Canada, do not exist. The Hamilton database was maintained by the Region’s Community Information Service—Information Hamilton. Most programs and services included in the database are run by not-for-profit organizations or government organizations. For-profit services were included if there were no not-for-profit agencies available that offered the same or similar service (eg, home oxygen providers). All database entries were tagged with keywords using the AIRS/211 LA Taxonomy of Human Services which is the industry standard for the Alliance of Information and Referral Systems (AIRS) [31]. To link the database to GENIE, keywords related to all topics of interest listed in the GENIE questionnaire were identified. For example, if a patient had an interest in walking or outdoor activities, programs and services were tagged with the AIRS categories of walking programs, walking tours, and nature centers or walks. This enabled GENIE to filter information from the database to match the selected areas of interest. Information Hamilton staff pulled together a complete list of all database items with selected search terms for our review for relevance to this adult population and to mark services for exclusion, such as youth programs or programs outside of the city. The database was updated daily by Information Hamilton’s staff, who regularly reach out and work with local service organizations to keep the database up to date.

#### Implementation of GENIE

GENIE facilitators were volunteers who attended a half-day training session to learn about the application of the GENIE tool during home visits. They visited with patients in 2 instances (the GENIE visit in Figure 1). At baseline, they would sign up patients and log on to GENIE, identify their social networks, and help patients to explore links to community support. The volunteers were trained to facilitate the use of the GENIE tool to engage in a discussion with the patients about their social networks, to discuss the access and use of community-based services, and to identify any additional services that they would like to access through the preparation of the GENIE report. The reports were sent to the patients’ electronic medical records for review by the primary care huddle team (a component of the Health TAPESTRY [Teams Advancing Patient Experience: Strengthening Quality] program) for planning and care coordination. The team told patients about any critical information regarding their plan of care and consulted with the relevant family physician in the clinic when needed. The report was printed and left with the patient after the home visit. After 3 months, the volunteer would return to the patient’s home, log on to GENIE, repeat the social network mapping, and revisit the GENIE report to determine if the patient had explored any HSSs. The volunteer role was intentionally limited to the role of a nonmedical volunteer facilitator. If any health care–based issues or concerns arose, patients would be encouraged to connect with their primary care provider [32].

### Setting and Sample

The study was conducted between August 2017 and March 2018 at 2 sites of a family health team (composed of 2 interprofessional primary care team clinics) that serves 30,000 patients in Hamilton, Canada. It targeted patients who were enrolled in Ontario’s Health Links Program [28]. Modeled after accountable care organizations in the United States, England, Australia, and New Zealand, the Health Links Program was launched in 2012 in Ontario, Canada, to improve care coordination for patients with complex needs who are the highest users of the health care system. The program connects them to primary care providers and engages them in their health care via active care planning [28]. These patients are considered high-cost, high-need users of the health care system—the top 5% of the population who use two-thirds of the health care spending [33]. GENIE was implemented with a small sample of Health Links patients within the context of the Health TAPESTRY program. Health TAPESTRY provided a unique structure for GENIE’s implementation within a primary care setting, as the program includes the use of trained volunteers as an extension of the primary care team, who visit patients in their homes. Health TAPESTRY is a multicomponent primary care intervention that centers on supporting older adults’ life and health goals [34,35]. The Health TAPESTRY program components include (1) trained volunteers visiting in pairs to collect health information using web-based surveys related to health risks and life goals [36], (2) care coordination by an interprofessional primary care team, (3) the use of technology to share health information between volunteers and primary care providers, and (4) support for system navigation [37]. Trained community volunteers implemented the GENIE tool, in addition to the Health TAPESTRY surveys. GENIE results and survey data were compiled into a web-based report that was transmitted to the interprofessional primary care team to support the formulation of a patient care plan.

Ethics approval was received for this study from the Hamilton Integrated Research Ethics Board Project number 13-366. All participants provided written informed consent before data collection.

### Data Collection

GENIE was field tested for feasibility and usability with a small group of volunteers and patients who were also receiving the Health TAPESTRY program. Data collection methods included (1) use statistics, (2) field observation in the community with patients and volunteers, (3) field observation notes taken during primary care team meetings, (4) focus groups with clinicians and volunteers, and (5) patient interviews. A summary of the data collected is provided in Table 1, and the focus group guides are provided in Multimedia Appendix 1.

Field observations focused on the usability captured via researcher observations during home visits with volunteers and patients at baseline. Field usability testing [38,39] was conducted via observation to assess the cognitive processes of users performing task completion of the GENIE tool, as trained volunteers facilitated the use of the web tool in a home visit. LC and RV observed volunteer and participant dyads to identify potential usability problems, with particular attention to goal-action sequences and interactions between volunteers and participants, dimensions of competencies (skills and knowledge required to complete the tool), barriers to the productive use of the tool, time to complete the tool, and ease of use for participants and volunteers [39]. Field observation notes captured procedures for each step or task to complete the tool (Multimedia Appendix 2). Tasks included setting up, completing the introductory and demographic page, completing network mapping and the questionnaire, discussing and tailoring the results, and printing the final results and the *my network* page that included the social network map. It also included comments and questions raised by the participants and volunteers as they worked through the task. Field notes were recorded by LC at interprofessional team meetings, in which patient GENIE reports were reviewed and discussed.

**Table 1:** Data collection by participant type.

Participant type	Usability	Feasibility	Perceived outcomes
Patients	Use statistics (baseline; 3 months) Field observation during patient home visits by a pair of volunteers (time 1)	Use statistics (time to complete the GENIE^a^ tool) Interviews (3 months)	Interviews (3 months)
Volunteers	Field observation during patient home visits by a pair of volunteers	N/A^b^	Focus group (3 months)
Primary care providers	Monthly huddle (care coordination team meeting) notes Focus group (3 months)	Monthly huddle notes Focus group (3 months)	Focus group (3 months)

^a^GENIE: Generating Engagement in Network Involvement.

^b^N/A: not applicable.

RV and LC conducted focus groups with volunteers and clinicians from 2 teams immediately following their huddle team meetings, which helped to gain participation in the research. Semistructured interviews were conducted by LC with study participants to explore the feasibility of GENIE using an interview guide that applied concepts from the Normalization Process Theory (NPT) [40] and perceived impacts. The NPT has been used for the feasibility study of a web-based program in primary care [41]. All focus groups and interviews with providers took place 6 months after the first use of GENIE by patients. The interview and focus group guide were tailored for each participant group (patients, volunteers, and the primary care huddle team) to explore the feasibility, usability, and perceived impacts of GENIE (refer to Multimedia Appendix 1 for the full guides).

The participants were recruited via convenience sampling. Lists of Health Links patients were distributed to their physicians from the 2 clinics that participated in the Health TAPESTRY program. A total of 25 Health Links Program patients were invited to participate in the study via a letter from their primary care physician. Physicians selected these patients based on age (55-69 years), enrollment in Health Links, and a clinical assessment indicating that they could benefit from improved care coordination offered by the GENIE and Health TAPESTRY. The target number of participants was 10, with diverse demographic characteristics (gender and age), which was deemed sufficient for usability testing [42,43]. A research coordinator received signed consent forms from 11 potential participants who were contacted by telephone to schedule the first of 2 home visits. One participant could not be contacted, another participant died before the first planned visit, and a third patient participated in the first home visit but withdrew from the study because of mental health distress. A total of 8 participants completed the study. All clinicians involved in the Health TAPESTRY program were invited to participate in the focus groups, which included questions about the GENIE tool. All *huddle* clinicians (a selected small interdisciplinary core team who met regularly to plan patient care) participated in a focus group held at each of the 2 sites (n=16). The remaining clinicians who worked in the clinic and were members of the huddle team were also invited to participate in one-on-one interviews. Of all the clinicians, 17% (7/41) agreed to participate. All clinic managers and volunteer coordinators agreed to participate in the interviews (n=3). Three volunteers who were trained to facilitate the GENIE tool participated in the focus group.

### Participants

We recruited 5 male and 3 female patients (4 patients from each clinic) with an average age of 63 years (SD 4.6; range 57-69 years). Half of the patients (4/8, 50%) had no computer access and had never used computers, whereas the other half (4/8, 50%) had used computers regularly. A total of 6 patients were married, 1 was divorced, and 1 had unknown marital status. Participants had a mean of 3.9 (SD 1.8) chronic diseases, ranging from 2 to 8 chronic diseases, including depression (n=6), anxiety (n=5), diabetes (n=5), cancer (n=4), arthritis (n=4), chronic obstructive pulmonary disease (n=2), heart disease (n=2), and pancreatitis (n=1).

Of the 3 volunteers, 1 was a male university student and 2 were retired females. The primary care team participants were members of the interprofessional teams at 2 clinics. One team had 7 members and included a dietitian, an occupational therapist, a physiotherapist, a pharmacist, a system navigator, 2 physicians, and a registered practical nurse. The second team had 9 members and consisted of an occupational therapist, a physiotherapist, a pharmacist, a system navigator, a physician, a psychologist, a registered practical nurse, and 2 nurse practitioners.

### Analysis

Qualitative data (interview, focus group, huddle notes, and field observation notes) were uploaded and organized using NVivo (QSR International) software, version 10 [44]. Two authors (LC and RV) reviewed all the data sources. Field observation notes were coded in NVivo according to their organizing criteria using a qualitative descriptive approach (Multimedia Appendix 2) by LC, and they were reviewed by RV. LC coded the interview and focus groups by inductively organizing the coding using the interview guide that was guided by NPT [40]. RV reviewed all the coding, and the research team reviewed the final coding structure with themes to increase rigor in the results.

Participants’ network maps were categorized using criteria developed by the GENIE founders and coauthors AR and IV, to identify the network typologies (based on the size of the network and type of network members). All authors reviewed and discussed the preliminary and final findings and interpretations.

## Results

### Areas of Interest and Services Chosen for Potential Follow-up in GENIE

All patients completed a network map and a questionnaire that asked about a person’s interests in various types of activities. Table 2 shows the categories and areas of interest chosen by participants. Patients were most interested in getting more physically fit (8/8, 100%), managing their weight (6/8, 75%), and learning about their health condition (6/8, 75%). All other topics garnered some interest, except knowing more about supports for a pet. Participants chose a range of 4-9 topics of interest, with an average of 6.

Patients favorited programs or services from the HSS database for potential follow-up that were related to the following: social clubs (5/8, 62%), home support (2/8, 25%), swimming (2/8, 25%), drama and music (1/8, 13%), and fitness and exercise (1/8, 13%). A total of 4 patients followed up with their favorited community services, including community or seniors’ social clubs (n=3) and an aquafit class (n=1). One patient was disinterested in the community services. Patients could also find relevant HSSs to address the health conditions of interest. Patients selected diabetes (1/8, 13%), cancer (1/8, 13%), and other health conditions (1/8, 13%) but did not follow up on any of the relevant HSSs.

**Table 2:** Patient responses to GENIE (Generating Engagement in Network Involvement) survey questions related to topics of interest.

Categories and GENIE survey questions and subquestions			Patients with an interest in the category, n (%)
**Activities**			
	I am interested in doing creative things (subquestions include reading and writing, drama and music, and arts and crafts).	5 (63)	
	I would like to know more about social activities (social clubs).	4 (50)	
**Health**			
	I would like to learn more about my health (draws from a checklist of health problems including heart problems, diabetes, arthritis, kidney problems, cancer, anxiety, depression, hypertension, and other).	6 (75)	
	I would like to manage my weight better (subquestions include weight management and nutrition).	6 (75)	
	I would like to get more physically fit (subquestions include fitness and exercise classes, walking or outdoor activities, and swimming).	8 (100)	
**Learning**			
	I would like to know more about looking after someone (caregiving).	4 (50)	
	I would like to learn new skills or take a course.	2 (25)	
**Support**			
	I would like to see people more often (subquestions include friendly visiting, counselling, and caregiver support).	4 (50)	
**Independent living**			
	I would like to know more about things that will help me remain independent (subquestions include transportation services and financial and benefits advice).	5 (63)	
**Volunteering**			
	I would like to help other people (subquestion includes volunteering opportunities).	4 (50)	
**New addition**			
	I would like to know more about supports for my pets.	0 (0)	

### Feasibility and Usability

#### Overview

Most patients and volunteers shared positive perceptions of the tool overall, such as the perception that the tool was easy to follow and understand. In addition, most patients noted that they would recommend it to others. However, several feasibility and usability issues were identified, as listed in Textbox 1 and described later. Quotations from participants are indicated by the participant type and ID number, such as patient 1, volunteer 3, and clinician 5. As we did not always capture the clinician’s names in the huddle meeting notes, participant IDs are missing for some quotes.

**Feasibility and usability themes.**

**Feasibility: interview and focus group data, primary care provider huddle notes, and use statistics**

- Partial understanding of the purpose of GENIE (Generating Engagement in Network Involvement)
- Need for facilitation to complete GENIE
- Clinician's navigation ability superior to GENIE supported by volunteers
- Time to complete GENIE

**Usability: field observation notes**

- GENIE inputs
  - Challenges in completing sections of the tool related to terminology used and lack of clarity in instructions
    - Chronic disease terms not understood
    - Unclear questions in the questionnaire
    - Unclear instructions related to who to add to the network and labeling their relationship
- GENIE and database outputs
  - Limitations in the quality and quantity of health and social service results
    - Quality of data insufficient in relation to community resources to match a health and social services to a patient
    - Quantity of data creates information overload
  - Technological challenges
    - Email setup concerns
    - Challenges printing results

#### Feasibility

##### Partial Understanding of the Purpose of GENIE

Generally, clinicians, volunteers, and many patients had a partial understanding of the purpose of GENIE. Most understood that it was meant to assess if patients were connected to the community; to determine their social support, including family, friends, and community-based resources during times of stress; and to find community resources to support them that match the patient’s goals and assist them with self-management. Patient 3 explained the following: “it’s more or less so [...] who you can contact to help you with different things to achieve your goals.” Furthermore, a provider explained:

My understanding was to sort of trial this tool to help people find individualized supports for them, so individualized, as in based on their needs and hopefully in their local neighbourhood, supports that they identify that they need to accomplish the goals that they outlined for themselves.

Clinician 2

Some confusion was also evident. It was noted in an observation during patient 1’s home visit that volunteers needed to be clearer about the purpose of GENIE when explaining it to the patient. In addition, clinical teams required the research coordinator to explain how to interpret the maps and network typologies. Most importantly, there was a gap in understanding the link between the social network component of GENIE and the use of the network to help mobilize the uptake of HSSs.

##### Need for Facilitation to Complete GENIE

The need to have someone to facilitate the patient’s completion of GENIE was identified through many observational field notes. This was related to the lack of access to computers or the internet by some patients, as noted above. In addition, volunteers or the research coordinator needed to explain the purpose of the tool, including the network map and survey questions; read out the descriptions of the topics of interest; facilitate moving around in the network map; or support tailoring the selection of services for follow-up. Working through GENIE required assistance from the facilitator in all cases.

##### Clinician’s Navigation Ability Superior to GENIE Supported by Volunteers

This theme was raised by clinicians who believed that they were better able to tailor the service to their patients than what the volunteers could offer with GENIE. Clinician 4 noted the following: “The software is good, but it’s not as good as a person who actually knows what’s going on there.” Clinicians explained that they could better match patients to services, given their knowledge of their patients and services. Clinician 2 explained:

...sometimes that layer of information and that layer of referral is based on a depth of understanding of community services, a depth of understanding of the patient, and a depth of understanding of how they form relationships. And, you know, you are never going to copy that from a database.

The relevance of some selected HSSs was questioned by their clinicians. One clinician explained that going with the most convenient service based on location was not the best approach to select a service and that other criteria may be more important. On the other hand, another clinician questioned why a service was chosen by the patient that was out of the local area of the patient’s home. Furthermore, clinicians perceived that paper-based resources listing community services in the clinic, such as flyers, booklets, and posters, would be more popular for patients.

##### Time to Complete GENIE

GENIE was completed along with other screening surveys in the Health TAPESTRY program, adding time to the home visit. This created a challenge to completing GENIE, which took an average of 31.9 minutes (range 20-42). This time was in addition to the average of 57 minutes (SD 22) that the volunteers spent to complete the home visit. Volunteers also raised the challenge that, at times, completing GENIE broke the flow of the visit and took much longer than the numerous but short Health TAPESTRY surveys. Volunteer 2 noted:

...it delays things, they might lose interest while all this is still going on. It stops the flow of the visit.

This was noted as being less of a problem in completing the social network map than in completing the questionnaire.

#### Usability

##### GENIE Inputs—Challenges in Completing Sections of the Tool Related to Terminology Used and Lack of Clarity in Instructions

Two main usability challenges were apparent in completed GENIE. These included (1) the use of unclear terminology and (2) confusion related to the instructions about who was to be added to the network map and how to label them. The first screen on logging in asked patients to check their chronic health conditions from a list of common conditions. On the basis of observations, it was noted that one patient and some volunteers were confused about certain chronic disease terms, including hypertension and chronic obstructive pulmonary disease, requiring simplification of these terms. Observation notes also indicated that one question in the questionnaire, “I would like help caring for other people” was misunderstood by one patient. One volunteer and patient also misunderstood the category of *finances and benefits*, as this item fell under the category in the questionnaire related to remaining independent.

Although network mapping was reported by most volunteers to be generally easy to use and visually helpful, clarification regarding the criteria used to determine who gets added to the network map and how to label the relationship was needed. Everyone struggled with the criteria needed to put someone into the network circles. The guiding question asks, “Who are the people close to you who help you with a long-term condition?” Some questioned whether this meant the people who could help or those who were most important to the patient but did not necessarily help. Clinician 6 commented:

And that could be geographically or that could be emotionally or support-wise. And I wonder if that were clarified a bit that might also help to find the purpose of the tool.

Furthermore, volunteers were unsure about how to label some network members, such as roommates, social groups, and nurse practitioners.

##### GENIE and Database Outputs: Limitations in the Quality and Quantity of HSS Results

Usability challenges, noted in field observations and clinician and volunteer focus groups, were identified. Some programs or services appeared to be missing or outdated regarding the quality of the community’s HSS database. A huddle note indicated that a clinician asked, “Why didn’t YMCA cancer support programs come up?” Further, patient 1 asked, “Why the Burlington Seniors Centre on New Street does not show up?” Another limitation was the lack of details for some services, such as costs to participate, which raised concerns about the limits of matching a patient’s interests based solely on traveling distance. A note from both team meetings indicated that clinicians objected to having *just a list* of services in the output and explained that what needs to happen is *matching the patient to the service*, in other words, better tailoring of the service to the patient’s needs and context.

The sheer quantity of results of HSSs produced by the GENIE report, particularly if there were many interests selected, was noted to be overwhelming by many volunteers and clinicians. Clinician 3 noted:

Sometimes providing the information is overload for patients and it doesn’t really go anywhere. Many people will come with, you know, I was given four pamphlets about stuff and I don’t know what to do with this and I don’t know how to connect with them or I don’t – which one am I supposed to choose for myself.

Furthermore, volunteer 1 explained:

When you give someone a huge list which often came up as this massive list, like, I can feel [the patients] getting discouraged. I would get discouraged.

Despite this challenge, the clinic teams appreciated the list of potential resources as a useful starting point. The paper-based list was also appreciated by some patients:

Sometimes it’s nice to have something on paper.

Patient 3

##### Technical Challenges

Some patients had no email address or had to use a family member’s email address to create an account and log on to GENIE. They were worried that sharing an email address would open them up to spam and unwanted follow-up emails. The researcher assured them in the visit that their email would not be shared with anyone and that there would be no follow-up email. The researcher could also create a new email address to enable them only to use the GENIE tool.

Several participants also had technical challenges in printing reports because of the occasional problem of generating the PDF for the report, which was a function in the tool. In addition, some general challenges associated with the printer included connecting the printer to the tablet because the researcher needed to use Wi-Fi or a hotspot to print from a portable printer that did not work in a few instances. Patients preferred to have a printed copy of the report.

##### Evaluation Impacts

Clinicians perceived that GENIE strengthened their understanding of their patients’ social networks through social network maps, and in one case, it moved the team to work with a patient who was identified as being socially isolated. Furthermore, clinician 5 described:

There was one that we looked at just recently where caregiver strain was a major issue for the person and yet all their ties were people that they care for, and that highlighted, I think, the real dilemma in that situation.

In a few cases, the use of GENIE sparked patient action, including reviewing more GENIE results after the visit and actively connecting more with family. For example, patient 8 noted:

It’s sort of a graphic that shows you how little you might have [been] involved with the people that are close to you. Maybe it’s sort of like almost visual cues that maybe I haven’t been as close as possible to my family. And I think that also sparked me to be more active phoning them.

Participants from all groups identified several potential impacts of using GENIE. There was a perception that GENIE provided the opportunity to reduce isolation by encouraging and enabling patients to become more involved in their community and establish connections. For example, volunteer 3 noted that GENIE could potentially help patients “be more involved in community and connections with other people rather than just be isolated in their homes, perhaps as an encouragement to develop or maintain those connections.” Patient 8 explained how it could help clinicians gain a better understanding of the patient’s needs:

I think it does help people identify what they need. And then for the doctors or practitioners or whoever is there that it’s just one more thing that, [...] sometimes you don’t have time to talk about everything when you go to your family doctor. So, I think it’s a good thing because support is really important to your well-being. And you had quite a few different things; [...] it was your medical plus the activities or social life.

There were limited changes in network size, frequency, or makeup over time. Of 8 patients, 4 had network membership gains at time 2 (3 months later), with new members added to their network as they joined clubs or classes. However, 1 patient experienced a network loss of 1 member. Patients did not note any changes in the frequency of contact with their network members or groups in the 3 months’ time frame (daily, monthly, weekly, or less). Finally, 25% (2/8) of patients showed no change in their network size or composition. Individual’s network typologies indicated that 50% (4/8) of patients had *very isolated* networks and 50% (4/8) of patients had *friend and family supported* networks at time 1. None of the patients had diverse networks at any time point.

### Factors Influencing Community Services Follow-up

Several barriers to the use of community services were identified. The most common barrier reported by patients and a volunteer was the distance to travel to the service or program. Patient 4 explained “I drive but I do not go downtown, and I do not like to drive downtown, and I do not like to rely on people for that.” Transportation issues were also noted, indicating that proximity is an important factor influencing service use. A few patients also mentioned that they did not want to leave home because of feeling unwell from chronic headaches or fear of getting confused owing to an acquired brain injury.

Motivation was identified by the clinic teams as a barrier for patients to access community-based services. On the basis of an observation, a patient identified key areas of concern as weight gain, information about his health, walking and outdoor activities, and caregiver support; although he seemed interested in these areas, he was not interested in attending any services or programs. Volunteers spoke about the potential of GENIE to influence motivation:

It creates awareness for people of what is available out there. And if they are motivated to do it, you know, that just might be the final push, if you will, to go out and do that.

Volunteer 1

Other barriers that were raised in a few instances were mobility issues, eligibility challenges (may not be eligible for transportation support because the patient can walk), and a lack of services in the area of interest.

## Discussion

### Principal Findings

This study showed that GENIE—a web-based social network tool—was generally feasible and usable for patients, volunteers, and the primary care team, although a number of feasibility and usability challenges were identified. A key feasibility challenge was the gap in understanding the purpose of GENIE by clinicians, patients, and volunteers. NPT points out that sense-making work, that is, having a clear understanding of the purpose of the novel intervention, is important to support its normalization or uptake in practice [40]. In this study, volunteers indicated that the social network maps were useful to help patients reflect on their personal support, and providers found it valuable to better understand their patients’ social contexts. However, there was a lack of understanding of how mobilizing the patient’s social network could help patients to access their community HSSs of interest identified through GENIE. Research has shown that social support can influence chronic disease self-management [45,46]. In a GENIE study involving older adults with diabetes, facilitators used GENIE as “a positive disruption to self-management by prompting reconsideration of network members and how they impact on self-management as well as an avenue to connect to new activities and sources of support” [47]. Given the partial understanding of the purpose of GENIE, this study highlights the need to improve GENIE training to ensure that users have a clear understanding of its purpose and how the components are meant to work together.

Another key feasibility challenge was that primary care providers reported relying more on their personal knowledge of HSSs than on GENIE results. In addition, NPT suggests that individually and collectively, participants need to see the value, benefit, and importance of the innovation for it to be taken up in practice [40]. Our results indicated that clinicians had a somewhat limited belief in the benefits of GENIE. They explained that their long-standing knowledge of their patients increased their ability to suggest suitable HSSs compared with GENIE search results. Other research has shown that physicians rely on their health care team’s knowledge of HSSs [20], use out-of-date resources to identify programs and services, and the HSS search strategies that are used are limited. Ploeg et al [48] also found making referrals to HSSs challenging. It has been shown that physicians understand the importance of social care needs; however, they do not have sufficient time to address them, necessitating assistance from others to fill this gap [49]. Physicians have relied on the interprofessional team to make community linkages for their patients [20], adding pressure on the team to be up to date on HSSs. This is a particular challenge in primary care practices that do not have interprofessional team support. Even when interprofessional teams are present, it takes time to identify the patient’s needs, match them to relevant services, and assist them in making the necessary connections. Additional time is required to consider patients’ personal social networks and mobilize them to assist in HSSs uptake.

It was not surprising that the time needed to complete GENIE was a feasibility challenge, given that it was used within the Health TAPESTRY program, which was time-intensive on its own. This is the first instance to our knowledge that GENIE was implemented in the context of another program—Health TAPESTRY. As such, it was not possible to determine the feasibility issues of GENIE, independent of the context of Health TAPESTRY. However, the Health TAPESTRY program provided the necessary infrastructure for GENIE to be implemented in a novel way by volunteers visiting patients at home within a primary care context, thereby adding new knowledge to the field.

Usability results showed that better GENIE filtering of HSS results was needed (eg, costs of services and eligibility criteria). Patients found the number of HSS results from GENIE overwhelming, particularly when the patient indicated interest in multiple activities. This generated numerous pages of results. Given this usability challenge, it is not surprising that facilitation was needed to support the use of GENIE to help prioritize the selections. Consistent with previous studies, there was an awareness of the need to focus on a narrower set of options. The key role of conversations with the facilitator was to understand acceptable options for new activities and training facilitators to understand and develop their role in helping the patient negotiate a focus on a small number of options acceptable to them [26,50]. Furthermore, improved facilitator training was needed to guide the participant to place themselves “at the centre of the circle and encourage them to think about why and how some people and resources might be more or less important to them” [50] to help HSSs uptake.

The accuracy and completeness of the database of community services that was feeding GENIE’s output and GENIE’s filtering capability also had some usability challenges. For example, a patient may have indicated an interest in a swimming program; however, the listing would not indicate its relevance to seniors. Future research is needed to better identify what patients want from an HSS database to help them further refine their results and identify relevant HSSs to meet their needs. Although it has been argued that there is a need for comprehensive and centralized community information systems [51], keeping information and referral database content up to date is challenging [52]. Community information and referral service databases in the United States and Canada tend to be managed by libraries or other community service agencies offering formal and structured community information services. Community capacity to maintain these databases requires consistent government funding. The research team worked closely with the regional community organization to notify them about missing or irrelevant information, which helped keep their databases up to date. Creating an ongoing feedback loop between primary care providers, volunteers, and community agencies who manage community service information databases could be a useful strategy to help increase their accuracy and increase the relevance of the data shared. Another potential solution would be to allow primary care providers to leave comments on HSS databases based on their knowledge and feedback from their patients on the HSS.

Usability results showed a need for modifications to the tool and more volunteer training to clarify the use of terms and phrases. For example, the use of the phrase *someone close to you* was a point of confusion between the volunteer and the patient in their interaction to map a patient’s personal network. A second important usability finding was that a few patients had difficulties understanding medical terms for chronic conditions. This indicates a need to strengthen health literacy for some adults with chronic conditions particularly, as it has been shown to influence the appropriate use of health services [53]. Future research is needed to better address the gap between the use of professional terminology and the public’s understanding when used in eHealth tools [54].

A systematic review by Stellefson et al [55] found that multidisciplinary teams (eg, diabetes care managers, nurses, primary care physicians, pharmacists, and social workers) can support patients’ use of the web 2.0 interventions to assist in the management of chronic diseases. This review supported the need for facilitation in the use of digital tools for self-management among older adults, which may be particularly important for those who are managing multiple chronic health and social conditions and are high users of the health care system. Furthermore, studies have shown that community members prefer to receive information informally through everyday conversations rather than via databases and that having skilled people in the community helps to translate information for them [56]. Finally, obtaining information from web sources can be challenging for adults with sensory losses, language issues, or poor health literacy, requiring facilitation by others [51]. This study supported that trained volunteers were a valuable extension of the primary care setting that could support the use of GENIE by high users of the health system. However, improvements are needed to (1) the GENIE app and the HSSs database, (2) provide a stronger orientation of the benefits of GENIE to clinicians, and (3) deliver more comprehensive training for volunteers. NPT speaks of the importance of allocating the division of labor around skill sets to support operationalizing the innovation [40]. Ideally, volunteer training would expand the volunteer role from that of a basic facilitator to include skills in motivational interviewing and increasing their knowledge about HSSs. The primary care team would need to endorse this enhanced volunteer role. Overall, more research is needed to explore how to better engage primary care in mobilizing HSSs support for these vulnerable adults.

With regard to outcomes, the patient uptake of services that were identified by GENIE was variable. Half of the patients (4/8, 50%) attended a new club or social group as a result of the intervention, pointing to the need and interest in social connection. A 2017 systematic literature review of social prescribing in primary care indicated positive impacts, including the use of recommended community links among others [57]. However, their results should be considered with caution, as the included studies were shown to have a high risk of bias. This feasibility study involved high users of the health care system and therefore likely presented participants with significant barriers to HSS uptake. More research is needed to explore which populations can benefit the most from such interventions and whether the addition of social prescribing by primary care providers combined with the use of GENIE supported by volunteers as an extension of the team would result in the better uptake of HSSs. Finally, 2 perceived outcomes of GENIE were identified in our study that can inform outcomes in future research with GENIE—the reduction of social isolation and the increased awareness of patients’ needs, interests, and social context among their primary care providers.

Finally, the most common topics of interest selected by patients were health related: weight management, physical activity, and health management. These topics mirrored those of the top 6 life and health goals identified by older adults in another study conducted in Ontario [32]. Furthermore, GENIE studies conducted in the United Kingdom also showed that most activities chosen in other related GENIE studies also tended to be health related [26,27]. This provides support that the topics in the GENIE tool are relevant to an adult population. However, it should be noted that patients were not offered the opportunity to nominate other topics of interest.

### Strengths and Limitations

This study involved 8 patients who were high users of the health care system, a population that is not frequently studied. A strength of this study is that data were collected from multiple sources (observation, interviews, focus groups, and GENIE use statistics) and from the perspectives of patients, providers, and volunteers to assess the feasibility, usability, and perceived impacts of GENIE. There were challenges in recruiting and retaining patients, and as such, the study is limited by its small sample size. However, the size is adequate for usability testing [42]. Data were collected between 2017 and 2018. Although technology rapidly changes, GENIE’s main functions remain relevant in addressing current challenges in primary care. In addition, the use of volunteers in primary care is a more recent innovation that continues to be tested [58], and we believe that this study is relevant today. Moreover, the implementation of GENIE occurred in the context of the Health TAPESTRY program. In this regard, the transferability of the study findings is low. Despite this, the study has contributed new knowledge about the feasibility and usability of using GENIE in a primary care context that involves trained volunteers as a unique extension of the team. Finally, as with most complex interventions, more research is needed to isolate and identify factors influencing the success and failure of its implementation and outcomes.

### Conclusions

GENIE provides an opportunity for patients to identify a program or activity that could help expand their social network and to identify social support that can be leveraged to increase social participation. More volunteer training and experience were required to enhance the implementation of GENIE to its full potential. Over time, volunteers may develop more familiarity with the landscape of HSSs and play a more important role in navigating patients through the system. With well-trained and experienced volunteers, more active follow-up (by phone or in-person), and perhaps actual accompaniment to attend a new program or service, more success might be possible in matching patients to programs and services. However, some clinicians perceive that there can be a mismatch in the right services for patients through GENIE. Volunteers, or perhaps peer approaches, may be a viable solution to support social prescribing and the uptake of services for populations living with complex health and social conditions needing self-management support. This study informs potential measures of research outcomes and points to GENIE’s potential. More research is needed to investigate the impact of one-on-one facilitation in primary care by volunteers and digital tools, preferably using comparative designs [56] that consider cost.

## Appendix

Multimedia Appendix 1 Interview guides for GENIE (Generating Engagement in Network Involvement).

Multimedia Appendix 2 GENIE (Generating Engagement in Network Involvement) observation field notes.

## Abbreviations

AIRS: Alliance of Information and Referral Systems
GENIE: Generating Engagement in Network Involvement
HSS: health and social service
INSPIRE-PHC: Innovations Strengthening Primary Health Care Through Research-Primary Health Care
MCC: multiple chronic condition
NPT: Normalization Process Theory
TAPESTRY: Teams Advancing Patient Experience: Strengthening Quality
