# Supplementary material for: A Web-Based Social Network Tool (GENIE) for Supporting Self-management Among High Users of the Health Care System: Feasibility and Usability Study
Source: JMIR Form Res. 2021 Jul 12;5(7):e25285. doi: 10.2196/25285 (PMC8315309; doi:10.2196/25285)
Supplement: Multimedia Appendix 1 [file formative_v5i7e25285_app1.docx]

# Appendix 1: Interview Guides for GENIE

I**nterview / Focus Group Guide for HEALTH CARE PROFESSIONALS based on Normalization Process Theory**

First we will ask some questions about what you liked and didn’t like about **GENIE, within the context of the Health TAPESTRY program**. When we refer to the TAPESTRY program, we are talking about the use of volunteers to visit clients in their homes, the collection of client data resulting in the TAP report plus the GENIE report, the work of the interprofessional teams’ to respond to the TAP reports, and the use of technologies (the Tap App, OSCAR, and the PHR) to share information and communicate across the healthcare team (volunteers, clients, healthcare professionals). When we refer to GENIE we are talking about the social network map (the three circles and the people names on it), and the questionnaire to assess client’s areas of interest, which then produces a list of health and social services and resources in response to the questionnaire.

For all of the questions below, we really want to know how GENIE has made a contribution or not to the work you do with the TAPESTRY program. We will ask you how you used the TAPESTRY report with the addition of the GENIE report, and if and how you worked it into your usual routine. Finally we will ask you about impacts you feel the TAPESTRY report with the addition of the GENIE report has had, and any recommendations you have for us to improve the GENIE tool itself, and ways of using it.

When we refer to the **team**, we mean the client, volunteers and other health professionals in the practice.

1. What do you think is the goal or purpose of GENIE?
2. What worked well and what did not with the GENIE report?

[Prompts: ease in understanding the results on completion; relevance or appropriateness of the questions; time to complete the tool or review the GENIE TAP report]

1. How does GENIE differ from your current ways of working (to obtain /share information about your client’s personal networks, and community-based based social and health care resources)?

[Prompts: How was GENIE or its results used in client or clinical encounters? When and how did you refer to it during the huddles?]

1. What new or different information does the GENIE report provide, compared to the standard TAP report, and how useful is it?

[Prompts: How useful was the personal mapping tool in clinic huddles? Did it contribute valuable new information? How did this new information help you (or not)?]

[Prompts: How beneficial was it to get more information about health and social services, or to help to identify HealthLinks patients’ networks?]

1. What do you understand are your tasks and responsibilities in relation to GENIE?
2. Reading the report
3. Responding/following up on the Report and by what mechanisms (volunteer visits, PHR communication, phone call to client, etc.)
4. What do you think are the drawbacks or threats in using GENIE?

[Prompts: what do you think about the availability of resources? What are your thoughts about sharing information about people’s networks?]

1. What actions or procedures (if any) have been created for or by you and/or the team to use GENIE and to continue using it? And what is missing?

[Prompt: What key pieces of information need to accompany the tool and why? What was missing, if anything?]

1. What do you think is the value in GENIE? In what ways if any did GENIE help you:
   1. in caring for adults over 55 years of age with complex health and social conditions (HealthLinks patients)?
   2. in working with the interprofessional team (healthcare professionals, clients, volunteers)?
   3. in meeting the client’s health and life goals?
2. How have you been able to make GENIE work for you and the team (clients, providers, volunteers)?

How do you and the team integrate GENIE into the existing TAPESTRY program?

What do you think about the integration of GENIE into the existing TAPESTRY program?

[Prompts: Is the work allocated evenly and appropriately? Did GENIE require additional resources and are these adequate? What kinds of resources are available to support GENIE and what do you think of them? ]

1. How much confidence do you have in GENIE for it to work for you and the team (clients, healthcare professionals, volunteers)? Explain.
2. Did using GENIE have any negative consequences or harms to you, clients or the team?

[Prompt: Did you find the information collected was repetitive or incongruent with other assessments and led to confusion? Did you ask questions that you could not act on to respond?]

1. What needs to be changed or improved with the use of GENIE to be more impactful or effective?

[Prompts: Any changes to the Web application, the report, or other aspects?]

I**nterview / Focus Group Guide for VOLUNTEERS based on Normalization Process Theory**

First, we will ask some questions about what you liked and didn’t like about **GENIE, within the context of the TAPESTRY program**. When we refer to the TAPESTRY program, we are talking about your visits to clients in their homes, the collection of client data on the TAP APP, resulting in the TAP report plus the GENIE report, the work of the interprofessional teams’ to respond to the TAP reports, and the use of technologies (the Tap App, OSCAR, and the PHR) to share information and communicate. When we refer to GENIE we are talking about the social network map (the three circles and the people names on it), and the questionnaire to assess client’s areas of interest, which then produces a list of health and social services and resources in response to the questionnaire.

For all of the questions below, we really want to know how GENIE has made a contribution or not to the work you do with the TAPESTRY program. We will ask you how you used the TAPESTRY Application with the addition of the GENIE tool, and if and how you worked it into your usual routine. Finally we will ask you about impacts you feel the TAPESTRY program with the addition of the GENIE has had, and any recommendations you have for us to improve the GENIE tool itself, and ways of using it.

When we refer to the **team**, we mean the client, volunteers, and healthcare professionals involved in the TAPESTRY program.

1. What do you think is the goal or purpose of GENIE?
2. How easy was the GENIE tool to complete? Explain?

[Prompt: How does it compare to using the other tools in the TAP App?]

1. What worked well and what did not in using GENIE?

[Prompts: relevance or appropriateness of the questions; time to complete the tool, etc.]

1. What does the GENIE tool add to the “standard” TAPESTRY program (the TAP App without GENIE)?

[Probe: How did GENIE change the client visit compared to the “standard” TAP visit?]

1. What do you understand are your tasks and responsibilities in relation to GENIE?
   1. At the first patient visit
   2. In the follow up visit (at 6 months)
   3. In response to any requests for follow up from the clinic team based on the client’s GENIE report?
2. What do you think are the drawbacks or threats in using GENIE?

[Prompts: what do you think about the time needed to use it and the availability of resources? What are your thoughts about sharing information about people’s networks?]

1. What actions or procedures if any have been created for or by you and/or the team to use GENIE and to continue using it? And what is missing?

[Prompt: Training to use GENIE: What key pieces of information need to accompany the tool and why? What was missing, if anything?]

1. What do you think about the integration of GENIE into the existing TAPETSRY program?

[Prompts: Did the integration of GENIE with TAP make sense to you? Does GENIE require additional resources and are these adequate? What kinds of resources are available to support GENIE and what do you think of them? ]

1. What do you think is the value in GENIE? In what ways if any did GENIE help you:
   1. in caring for adults over 55 years of age?
   2. in working with the interprofessional team (healthcare professionals, clients, volunteers)?
   3. in meeting the client’s health and life goals?
2. What new or different information does the GENIE report provide, compared to the standard TAP report, and how useful is it?

[Prompts: How useful was the personal mapping tool in clinic huddles? Did it contribute valuable new information? How did this new information help you (or not)?

Prompts: How beneficial was it to get more information about health and social services, or to help to identify HealthLinks patients’ networks?]

1. How much confidence do you have in GENIE for it to work for you and the team (clients, healthcare professionals, volunteers)? Explain.
2. Did using GENIE have any negative consequences or harms to you, clients or the team?

[Prompt: Did you find the information collected was repetitive or incongruent with other assessments and led to confusion? Did you ask questions that you could not act on to respond?]

1. What needs to be changed or improved with the use of GENIE to be more impactful or effective?

[Prompts: Any changes to the Web application, the report, or other aspects?]

1. Do you have anything else to add?

**Time 1** I**nterview / Focus Group Guide for CLIENTS based on Normalization Process Theory (3 months after first visit)**

First we will ask some questions about what you liked and didn’t like about **GENIE, within the context of the TAPESTRY program**. When we refer to the TAPESTRY program, we are talking about the use of volunteers to visit you in your home, the collection of data on the iPad, resulting in the TAP report plus the GENIE report that goes to your doctor’s office, and the work of the interprofessional teams to respond to the TAP reports, and finally, the use of technologies (the Tap App, OSCAR, and the PHR) to share information and communicate. When we refer to GENIE we are talking about the social network map (the three circles and the people names on it), and the questionnaire to assess client’s areas of interest, which then produces a list of health and social services and resources in response to the questionnaire.

For all of the questions below, we really want to know how GENIE has made a contribution or not to your experience of the TAPESTRY program. We will ask you about any recommendations you have for us to improve the GENIE tool itself, and ways of using it.

When we refer to the **team**, we mean you, the volunteers and the healthcare professionals involved in the TAPESTRY program.

1. How easy was the GENIE tool to complete today? Explain?

[Prompts: How did you find completing the network map (the part with the circles)? How did you find completing the questionnaire? How does it compare to rest of the TAP App (the questionnaires about your health)? What worked well, what did not?]

1. How did your doctor’s office follow up on the results from the first visit we had three months ago?

[Prompts: Did you get a phone call from your doctor’s office about the TAPESTRY or GENIE reports? Did they mention anything about the TAPESTRY or GENIE reports when going for any appointments in the last six months?]

1. Have you used the GENIE tool or referred to the handout report since we were here last (3 months ago)? Please explain why or why not.

**Time 2** I**nterview / Focus Group Guide for CLIENTS based on Normalization Process Theory**

First we will ask some questions about what you liked and didn’t like about **GENIE, within the context of the TAPESTRY program**. When we refer to the TAPESTRY program, we are talking about the use of volunteers to visit you in your home, the collection of data on the iPad, resulting in the TAP report plus the GENIE report that goes to your doctor’s office, and the work of the interprofessional teams to respond to the TAP reports, and finally, the use of technologies (the Tap App, OSCAR, and the PHR) to share information and communicate. When we refer to GENIE we are talking about the social network map (the three circles and the people names on it), and the questionnaire to assess client’s areas of interest, which then produces a list of health and social services and resources in response to the questionnaire.

For all of the questions below, we really want to know how GENIE has made a contribution or not to your experience of the TAPESTRY program. We will ask you about any recommendations you have for us to improve the GENIE tool itself, and ways of using it.

When we refer to the **team**, we mean you, the volunteers and the healthcare professionals involved in the TAPESTRY program.

1. **OVERALL UNDERSTANDING**
2. What do you think is the goal or purpose of the Health TAPESTRY program?

[Probe: What do you think is the goal or purpose of TAPESTRY?]

1. Thinking about the GENIE tool specifically (the personal network map with the circles, and the questionnaire that produces the map of local community services), what do you think is the goal or purpose of GENIE?
2. **NORMALIZATION in PRACTICE**
3. How easy was the GENIE tool to complete today? Explain?

[Prompts: How did you find completing the network map (the part with the circles)? How did you find completing the questionnaire? How does it compare to rest of the TAP App (the questionnaires about your health)? What worked well, what did not?]

1. How did your doctor’s office follow up on the results from the visit we had six months ago?

[Prompts: Did you get a phone call from your doctor’s office about the TAPESTRY or GENIE reports? Did they mention anything about the TAPESTRY or GENIE reports when going for any appointments in the last six months?]

1. Have you used the GENIE tool or referred to the handout report since we were here last (6 months ago)? Please explain why or why not.
2. How does using GENIE differ from your current ways of getting and sharing information about important people in your life, and community based social and health care resources?

[Prompts: Did you share GENIE or its results with others and if so, with whom and why? Did you try out any services? Why or why not?]

1. **IMPACTS and OUTCOMES**
2. In what ways if any did GENIE help you:
   1. in meeting your life and health goals?
   2. in managing your health condition(s)?
   3. in communicating and collaborating with your health care team (volunteers and health care providers)?
   4. In finding out about programs and services in your local community?

[Prompts: How beneficial was it to get more information about health and social services, or to help to identify your network?]

What did you think of the care plan? (design, content, format, usefulness)

1. What do you think needs to be changed or improved for GENIE to be more impactful or effective?

1. How has the TAPESTRY program affected how you are able to manage your health?
2. Is there anything else you would like to add?
