# Supplementary material for: A Web-Based Social Network Tool (GENIE) for Supporting Self-management Among High Users of the Health Care System: Feasibility and Usability Study
Source: JMIR Form Res. 2021 Jul 12;5(7):e25285. doi: 10.2196/25285 (PMC8315309; doi:10.2196/25285)
Supplement: Multimedia Appendix 2 [file formative_v5i7e25285_app2.docx]

**Appendix 2: GENIE Observation Field Notes**

GENIE Client #

Date:

Summary:

OVERALL TIMING

VISIT:

TAPESTRY Tools:

EU-GENIE:

USABILITY OF GENIE TOOL (goal-action-sequences)

| Goal | Note CLIENT procedure, requests, comments and nonverbal responses (body movements, gesture, gaze)  Note VOLUNTEER procedure, requests, comments and nonverbal responses (body movements, gesture, gaze) |
| --- | --- |
| Set up |  |
| Complete Introduction  And Demographic Page |  |
| Complete Network Mapping |  |
| Questionnaire Completion |  |
| Results Discussion and Tailoring |  |
| Closing  Print Results Page and My Network Page | -- |
| Comments |  |
